# The tear fluid mucin 5AC change of primary angle-closure glaucoma patients after short-term medications and phacotrabeculectomy

**Published:** 2010-11-09

**Authors:** Wei Liu, Huilin Li, Duanyang Lu, Juan Liang, Xiaoli Xing, Aihua Liu, Shaozhen Zhao, Xiaorong Li, Jian Ji

**Affiliations:** 1 Tianjin Medical University Eye Center, Tianjin, China; 2 Eye Department of Changzhi Medical College, Shanxi Province, China; 3 Eye Department of Luoyang Center Hospital, Henan Province, China

## Abstract

**Purpose:**

This paper proposed to evaluate the tear fluid mucin 5AC (MUC5AC) change in Chinese primary angle-closure glaucoma and cataract patients after short-term medications and phacotrabeculectomy.

**Methods:**

Twenty-five eyes of 25 consecutive Chinese patients with coexisting visually significant cataract and angle-closure glaucoma and 40 eyes of 40 volunteers enrolled in this study were investigated. Tear fluid from normal subjects and patients (1 day pre-operatively, 1 month, 3 months, and 6 months post-operatively, respectively) were collected. The MUC5AC protein levels in the tear fluid were determined by Enzyme-Linked ImmunoSorbent Assay (ELISA). The MUC5AC change after phacotrabeculectomy was evaluated.

**Results:**

The MUC5AC quantity of the patients after short-term medications was 16.95±12.86 ng/ml, compared with 32.39±18.44 ng/ml MUC5AC quantity of the controls. There was a significant difference between the two groups (p<0.05). The MUC5AC of the patients decreased significantly to 6.91±7.11 ng/ml at 1 month after surgery (p<0.05). At 3 months after surgery, the MUC5AC recovered to 15.53±12.63 ng/ml, and had no significant difference with the pre-operative level (p=0.26). At 6 months after surgery, the MUC5AC was 18.94±14.64 ng/ml and had no significant difference with before-surgery levels (p=0.14).

**Conclusions:**

Both phacotrabeculectomy and short-term anti-glaucoma medications can decrease the MUC5AC in the tear fluid of primary angle-closure glaucoma patients. At 3 months after phacotrabeculectomy, the MUC5AC could recover to the pre-operative level. More attention should be paid to the tear film stability and ocular surface physiology in these patients.

## Introduction

The preocular film is composed of a thin outer layer of Meibomian lipids and an inner aqueous phase adjacent to the glycocalyx of the apical cells of the epithelium. Epithelial mucins, a family of high-molecular-weight glycoproteins, are the major protein components of the aqueous phase of the tear fluid [1,2]. Mucins have been ascribed several roles: maintaining a smooth surface for light refraction, lubricating the eyelids, the conjunctiva and the cornea, supplying the cornea with nutrients and removing foreign materials from the cornea and conjunctiva. Mucins can also participate in the defense of the ocular surface via specific and nonspecific antibacterial substances [3].

The ocular surface epithelium expresses at least four major mucin genes: MUCIN 1 (MUC1), MUCIN 4 (MUC4), MUCIN 5AC (MUC5AC), and MUCIN 16 (MUC16). MUC1, as a transmembrane mucin, is reported to be produced by the corneal and conjunctival epithelia [4,5]. MUC4, also a transmembrane mucin, is expressed by corneal as well as conjunctival epithelia. Although MUC4 is present in the cornea, the expression of *MUC4* mRNA has been demonstrated in limbal and peripheral cornea, with decreasing levels toward the central cornea [6,7]. MUC16, also known as the CA125 antigen, is also a membrane-associated mucin expressed by the ocular surface. MUC16 is a key component of the ocular surface glycocalyx, where plays a role in barrier formation on the surface of epithelial cells [8]. MUC5AC is a gel-forming secretory mucin expressed by conjunctival goblet cells. MUC5AC is the main mucin component of the tear film [7,9,10].

The negative effects of anti-glaucomatous medications on the ocular surface have been known from previous studies and reports. In short, ocular surface disease (OSD) is prevalent among medically treated patients with glaucoma; intraocular pressure (IOP)-lowering medications can contribute to OSD, in part due to the preservatives used in medications; the severity of OSD symptoms is positively correlated to the duration and number of IOP-lowering medications used [11-14]. However, there is few studies concerning the ocular surface change of glaucoma patients induced by surgery.

In the present study, we evaluated the quantity of MUC5AC in the tear fluid of patients with glaucoma combined with cataract after the use of topical anti-glaucoma medications for less than 6 months, and monitored the MUC5AC change of the patients after phacotrabeculectomy.

## Methods

### Patients and volunteers

This study adhered to the tenets of the Declaration of Helsinki. This study was approved by the ethics committee of Tianjin Medical University Eye Center, Tianjin, China. Written, informed consent was obtained from all primary angle-closure glaucoma (PACG) patients and volunteers. The diagnosis of PACG was made based on elevated IOP, a shallow anterior chamber, gonioscopic findings of synechia, glaucomatous optic disc cupping, and the Humphrey visual field defects. The inclusion criteria for the study group were: (1) PACG and coexisting cataract in the same eye; (2) anti-glaucoma eye drops administered for less than 6 months. The inclusion criteria for the control group were: (1) the company of the patients who were not administered any eye drops; (2) able and willing to give an informed consent to take the tear fluid sample. The exclusion criteria for both the study group and the control group included a history of disease that could affect the ocular surface (blepharitis, dacryocystitis, keratoconjunctivitis sicca, etc), contact lens wearing and previous ocular surgery or systemic disease, such as diabetes mellitus.

### Sample collection

Tear fluid from normal subjects and patients (1 day pre-operatively, 1 month, 3 months, and 6 months post-operatively, respectively) were collected to determine MUC5AC protein levels. At least 5 μl open eye tears were collected from the outer canthus of patients/volunteers using glass capillary tubes. No anesthetic or other medication was applied. The glass capillary tube was not allowed to touch either the lid or any part of the globe. The collected tear fluid was stored in −20 °C for further analysis.

### Enzyme-Linked ImmunoSorbent Assay (Elisa)

The rat anti-human Mucins 5ac Elisa kit (Diagnostic Systems Laboratories, Webster, TX) was used and the procedure was conducted as the introduction book indicated as follows. First, the standard curve was made using the standard samples (25, 50, 100, 250, and 500 pg/ml). Then 5 μl tear fluid sample was diluted to 1:40 with diluted sample dilution, and then 100 μl diluted samples were transferred into the appropriate wells. After incubation for 30 min at 37 °C,the wells were aspirated and washed 5 times with the diluted wash buffer; then 100 μl enzyme conjugate was added to each well. The incubation (37 °C for 30 min) and aspiration/wash procedure was repeated. Followed by incubation for 15 min at 37 °C, 50 μl Color Reagent A and Color Reagent B were added to each well. Finally 50 μl Stop solution was added to each well. The absorbance and the MUC5AC concentration of the solution in the wells were recorded within 15 min. The ELISA was performed in duplicates for each tear fluid sample and the average was recorded as the final result.

### Surgical procedure

All the patients accepted two-site phacotrabeculectomy performed by the same experienced surgeon (J.J.). The pupil was dilated with 0.5% tropicamide drops (Shuanghe Co., LTD, Beijing, China) 30 min before surgery. All the surgeries were made under peribulbar local anesthesia with 2% lidocaine (Jinyao Co., LTD, Tianjin, China). Ocular compression was performed in every case. A fornix-based conjunctival flap was fashioned at the 12 o’clock position. The 1/2 thickness rectangular scleral flap (3×4 mm) was performed with a crescent knife. A sponge soaked with 0.2 mg/ml mitomycin-C (MMC; Haizheng Pharmaceutical Co., Zhejiang , China) was applied under the conjunctival flap for 2 min, and the area was washed out with a balanced salt solution. A small limbal paracentesis was performed at the 2 o’clock position. The anterior chamber was then entered along the corneal tunnel with the 3.0-mm keratome at the 10 o’clock position. The chamber was immediately deepened with Viscoat (S.A. Alcon-Couvreur N.V., Rijksweg, Puurs, Belgium). Pupil stretching or synechiolysis were performed when necessary. After the continuous curvilinear capsulorhexis, the nucleus was removed with a standard four-quadrant, “divide and conquer” technique. An automated irrigation/aspiration apparatus (Alcon Laboratories, Inc., Fort Worth, TX) was introduced into the anterior chamber to remove the cortical remnants and to polish the posterior lens capsule. The intraocular lens (IOL) was placed in the capsular bag. The pupil was contracted with 0.01% carbachol injection (Bausch & Lomb Freda, Jinan, Shandong, China). The scleral flap was then dissected forward into the clear cornea. A trabeculectomy of 1**×**2 mm was performed with a 15° scalpel and Vannas scissors. A peripheral iridectomy was performed, which was basally large enough to be sure that the iris could not be visualized in the base of the trabeculectomy opening. The rectangular scleral flap was closed with two 10/0 nylon sutures at the apex angles. The conjunctival wound was closed with two 10/0 nylon sutures.

### Post-operative care and follow-up

Post-operative medications employed routinely are 0.3% ofloxacin drops (Santen, Osaka, Japan) six times per day, 1% fluorometholone drops (Santen) six times per day, and 0.1% diclofenac sodium drops (Xingqi Pharmaceutical Co., LTD, Shenyang, Liaoning, China) four times per day. All the drops were stopped after 4 weeks of treatment and steroids were tapered off during the 4 weeks. All the patients required no anti-glaucoma medications after surgery. The tear fluid of the patients was collected on months 1, 3, and 6 post-operatively.

### Statistical analysis

Data was reported as Mean±SD. All analyses were performed with SPSS (IBM, Somers, NY) software version 13.0. Independent samples *t* test was used for the comparison of age and MUC5AC between patients and volunteers. The χ^2^ test was used for the comparison of gender between patients and volunteers. Paired samples *t* test was used for the pre-operative and post-operative comparisons of MUC5AC. A p<0.05 was considered statistically significant.

## Results

### Patients and controls

Twenty-five consecutive Chinese patients with coexisting visually significant cataract and PACG were enrolled as the study group. Forty volunteers were enrolled as the control group. No subjects refused to enroll and none were lost to follow-up during the course of the study. There were no statistical differences between the two groups regarding age (67.4±10.1 years versus 65.0±9.8 years, independent samples *t* test, *t*=0.94, p=0.35) or gender (χ^2^ test, χ^2^=0.19, p=0.66).

### Pre-operative anti-glaucoma medications

All the patients were administered at least one of the four anti-glaucoma eye drops for less than 6 months before surgery: 2% Carteolol hydrochloride (Mikelan; Otsuka Pharmaceutical Co., LTD, China), 1% Brinzolamide eye drops (Azopt; Alcon), 0.2% Brimonidine Tartrate eye drops (Alphagan; Allergan, Mayo, Ireland), 1% Pilocarpine nitrate eye drops (Yongguang Pharmaceutical Co., LTD, Sanhe, Hebei, China). There were 3 patients administered 2% Mikelan alone, 1 patient Alphagan alone, 11 patients 2% Mikelan and Azopt, 2 patients 2% Mikelan and 1% Pilocarpine, 1 patient 2% Mikelan and Alphagan, and 7 patients 2% Mikelan and Azopt and Alphagan.

### MUC5AC quantity of the patients and controls

The MUC5AC quantity of the patients detected by ELISA was 16.95±12.86 (range from 1.36 to 43.79) ng/ml, and 32.39±18.44 (range from 4.05 to 73.16) ng/ml of the controls. There was a significant difference between the two groups (independent samples *t* test, *t*=3.66, p<0.05).

### MUC5AC change of the patients after phacotrabeculectomy

The MUC5AC changes after phacotrabeculectomy are shown in Table 1. The MUC5AC decreased significantly from 16.95±12.86 ng/ml before surgery to 6.91±7.11 (range from 1.11 to 27.37) ng/ml 1 month after surgery (paired samples *t* test, *t*=4.93, p<0.05). At 3 months after surgery, the MUC5AC recovered to 15.53±12.63 (range from 1.23 to 47.47) ng/ml, and had no significant difference with the pre-operative level (paired samples *t* test, *t*=1.15, p=0.26). At 6 months after surgery, the MUC5AC detected by ELISA was 18.94±14.64 (range from 1.30 to 49.66) ng/ml, and had no significant difference with the MUC5AC before surgery (paired samples *t* test, *t*=-1.54, p=0.14).

**Table 1:** MUC5AC after phacotrabeculectomy **(**ng/ml).

** **	**Preop**	**1 Month**	**3 Months**	**6 Months**
MUC5AC	16.95±12.86	6.91±7.11	15.53±12.63	18.94±14.64
*t*		4.93	1.15	−1.54
p		<0.05	0.26	0.14

The MUC5AC decreased significantly at 1 month after surgery and recovered at 3 months after surgery. In the table, Preop: pre-operatively; 1 month: 1month post-operatively; 3 months: 3 months post-operatively; 6 months: 6 months post-operatively.

## Discussion

In our study, by using ELISA, we have demonstrated a significant reduction of MUC5AC mucin protein content on the ocular surface of PACG patients who accepted short-term topical medication treatment. For MUC5AC, which is secreted solely by conjunctival goblet cells, this indicates that, even for a relatively short-term topical anti-glaucoma medication therapy, the goblet cells of the patients may be decreased considerably, numerically and/or functionally. One of the reasons for the reduction of MUC5AC is the preservatives used in the anti-glaucoma medications. Several studies have reported toxic side effects from anti-glaucoma drugs on the conjunctiva, especially if preservatives are used [15-17]. The toxic action of preservatives on the ocular surface has been widely demonstrated in vitro as well as in vivo, in both humans and animals [18]. Preservatives can decrease the stability of the precorneal film, directly or indirectly. They have a direct detergent effect on the lipid layer, resulting in increased evaporation. Preservatives can also decrease the density of goblet cells in the conjunctival epithelium, which will decrease the MUC5AC content in the tear, and indirectly, destabilize the tear film [19]. By new combined tools, Pauly et al. [20] detected a significant MUC5AC positive goblet cells loss induced by preservatives; Albietz et al. [21] also detected a reduced goblet cell density in dry eye receiving preserved topical agents. Another reason for the reduction of MUC5AC includes the multiple treatments of the patients in our study. Histopathological and impression cytology studies of the conjunctiva have demonstrated that topical preservatives can lead to inflammation, squamous metaplasia, and subconjunctival fibrosis in the conjunctiva and Tenon’s capsule [19,22]. These side effects are dose dependent and increase with frequency of instillation [18]. Moreover, the increasing preservatives concentrations can also induce an enhanced apoptosis rate of the conjunctival epithelial cells [20]. In our study, only 4 patients were administered single anti-glaucoma medication treatment; most of the patients (21/25) accepted multiple treatments. The multi-use of anti-glaucoma medication will increase the above dose dependent toxic action of the preservatives on the conjunctiva and lead to the decrease of MUC5AC level.

Rodriguez et al. [23] showed a decrease in goblet cell populations until the third month after LASIK by impression cytology, which was highly correlated with suction time. Li et al. [24] revealed significant reduction of goblet cell density and the presence of serious squamous metaplasia in the epithelial layer of the globe conjunctiva at 3 months after cataract surgery. In our study, we also detected a decrease of MUC5AC in the tear fluid after phacotrabeculectomy, which probably resulted from the conjunctival squamous metaplasia and the reduction of goblet cell density, for MUC5AC of the tear fluid is secreted uniquely by the conjunctival goblet cells. The reduction in goblet cell density can be due to several factors such as toxicity of MMC introduced during the surgery and post-operative topical medication, damage of the corneal and conjunctival nerves and the limbus stem cells, inflammation, or mechanical trauma produced by the surgery [23]. In this study, we found the MUC5AC recovered to the pre-operative level at 3 months after surgery. This might have resulted from an adapted response against the conjunctival aggression [6], for there are numerous typical MUC5AC-positive goblet cells outside the edges of the filtering blebs, proved by impression cytology [25].

As the main mucin component of the tear film, MUC5AC is believed to play an important role in attainment of tear stability and in prevention of drying on the ocular surface [3]. The decrease in the amount of mucin molecules on the ocular surface will affect the stability of the tear film and compromise the normal ocular surface physiology; patients will feel more sensitive to environmental factors because the eye’s protective function is weakened [1]. In our study, we demonstrated a significant decrease of MUC5AC in the tear fluid of PACG patients after short-term anti-glaucoma medications or phacotrabeculectomy, and this indicates that, for these patients, more attention should be paid to the maintenance or recover of their tear film stability and ocular surface physiology.
